# Repurposing a prostate tumor marker: elevated FPSA/TPSA ratio as a novel non-invasive biomarker for declining eGFR in male type 2 diabetes

**DOI:** 10.3389/fendo.2026.1905743

**Published:** 2026-09-11

**Authors:** Shihua Zhao, Honglei Hu, Xiaodong Zhao, Lei Zhang, Deying Niu

**Affiliations:** 1 Department of Endocrinology, Zibo Central Hospital, Zibo, Shandong, China; 2 Department of Endocrinology, Qingdao Endocrine & Diabetes Hospital, Qingdao, Shandong, China; 3 Department of Neurosurgery, Zibo Central Hospital, Zibo, Shandong, China

**Keywords:** diabetic kidney disease, estimated glomerular filtration rate, free-to-total prostate-specific antigen ratio, real-world data, type 2 diabetes mellitus

## Abstract

**Background:**

Diabetic kidney disease (DKD) lacks accessible, cost-effective biomarkers for early screening. Given the distinct clearance pathways of free prostate-specific antigen (FPSA, renally excreted) and total prostate-specific antigen (TPSA, hepatically metabolized), this study investigated the association between serum FPSA/TPSA ratio and estimated glomerular filtration rate (eGFR) in male patients with type 2 diabetes mellitus (T2DM).

**Methods:**

In this real-world cross-sectional study, 4,487 male patients with T2DM were retrospectively enrolled. eGFR was determined using the Kidney Disease: Improving Global Outcomes (KDIGO)-recommended Chronic Kidney Disease Epidemiology Collaboration (CKD-EPI) creatinine-cystatin C (Cr-CysC) equation. Multivariable linear regression and subgroup analyses were conducted to elucidate the independent effect of the FPSA/TPSA ratio on eGFR, with adjustment for age, prostate-specific diseases, and metabolic confounders.

**Results:**

Baseline TPSA levels remained stable across renal function stages (median 0.9ng/ml, P = 0.524), minimizing interference from macroscopic prostate pathologies. After adjustment for age and metabolic profiles, the FPSA/TPSA ratio exhibited a robust independent negative association with eGFR (β = −0.2, P < 0.001). This inverse correlation remained consistent across all clinical subgroups (P for interaction > 0.05), with a more pronounced predictive trend among elderly patients (≥ 65 years). Notably, our multivariable models also captured the chronic kidney disease “lipid paradox”, whereby higher triglyceride levels paradoxically correlated with preserved eGFR (β = 0.51, P = 0.018).

**Conclusion:**

An elevated serum FPSA/TPSA ratio is a robust, non-invasive indicator of declining eGFR in male patients with T2DM and may improve DKD risk stratification and early clinical warning without additional economic burden.

## 1 Introduction

Diabetic kidney disease (DKD) is one of the most prevalent and clinically burdensome microvascular complications of type 2 diabetes mellitus (T2DM). It is the leading cause of chronic kidney disease (CKD) and end-stage kidney disease (ESKD) worldwide (1, 2). With the relentless rise in the global prevalence of T2DM, the associated decline in renal function, together with the increased risk of cardiovascular events and all-cause mortality driven by DKD, has emerged as a major public health challenge (2, 3). Although recent comprehensive therapeutic strategies—particularly renin-angiotensin system (RAS) inhibitors, SGLT2 inhibitors, and GLP-1 receptor agonists—have significantly improved renal outcomes in patients with T2DM, the early recognition and precise risk stratification of DKD remain highly challenging (3–5). Currently, clinical practice predominantly relies on the urinary albumin-to-creatinine ratio (UACR) and estimated glomerular filtration rate (eGFR) to evaluate renal impairment. However, because of the well-recognized “blind spot” effect of serum creatinine and the susceptibility of UACR to various non-specific factors such as physical exertion and systemic infections, a substantial proportion of patients have already experienced irreversible and progressive deterioration of renal filtration function before significant laboratory abnormalities are detected (6–8). Consequently, identifying novel, sensitive, and stable biomarkers that can detect early renal function decline in patients with T2DM remains an urgent unmet need in the fields of nephrology and endocrinology (9, 10).

Prostate-specific antigen (PSA), a member of the serine protease family, is primarily secreted by prostate epithelial cells and is routinely used in clinical practice for the screening and risk assessment of prostate diseases (11). Circulating PSA predominantly exists in two molecular forms: total PSA (TPSA) and free PSA (FPSA). The FPSA/TPSA ratio has substantial clinical utility in differentiating between benign and malignant prostatic conditions (12, 13). However, a growing body of evidence indicates that circulating PSA levels are not exclusively a proxy for local prostatic pathology. Rather, its expression and systemic clearance can be intricately modulated by a myriad of systemic factors, including advanced age, obesity, insulin resistance, sex hormone status, chronic inflammation, metabolic dysregulation, and renal function. In the context of metabolic disorders, fluctuations in PSA levels may reflect complex interactions within the endocrine-inflammation-metabolic network, providing a theoretical foundation for exploring their potential clinical implications in diabetes and its associated complications.

Previous studies have indicated that PSA profiles in patients with T2DM may differ significantly from those in non-diabetic populations. These discrepancies are hypothesized to be driven by hyperinsulinemia, hemodilution secondary to obesity, alterations in circulating testosterone, and chronic low-grade inflammation (14–17). Furthermore, impaired renal function directly compromises the systemic clearance and distribution of various medium- to large-molecular-weight proteins and their binding complexes. Existing literature suggests a robust association between declining renal function and both elevated FPSA levels and an altered FPSA/TPSA ratio, whereas TPSA levels remain relatively unaffected by renal impairment because of hepatic clearance (18). This kinetic divergence implies that the FPSA/TPSA ratio reflects not only prostate biology but is also profoundly shaped by the interplay of renal filtration, metabolic activity, and systemic inflammation. Nevertheless, whether a stable and independent association exists between the FPSA/TPSA ratio and DKD in male patients with T2DM remains systematically underexplored.

Notably, the pathogenesis and progression of DKD are driven not solely by chronic glycemic exposure, but by a synergistic combination of hypertension, lipid dysmetabolism, chronic inflammation, oxidative stress, endothelial dysfunction, and coagulation abnormalities (4, 6, 19). The established links between PSA-related indices and inflammatory cascades, metabolic syndrome, and hormonal imbalances suggest that PSA might serve as a “crosstalk signal” bridging physiological prostate metabolism and renal metabolic injury (16, 20). Particularly among male patients with T2DM—among whom advancing age, benign prostatic hyperplasia (BPH), chronic inflammation, and renal decline frequently coexist—the FPSA/TPSA ratio is a routinely tested and widely accessible variable within electronic medical records (EMRs). If independently associated with DKD risk, this ratio could be repurposed as a highly scalable, low-cost supplementary tool for DKD risk identification (17, 18).

However, evidence characterizing the relationship between PSA subtypes and diabetic renal impairment remains exceedingly sparse. Most existing studies have focused disproportionately on the relationship between PSA and prostate cancer, metabolic syndrome, or overall kidney function in general populations (11, 18), leaving a critical gap in real-world evidence regarding the predictive value of the FPSA/TPSA ratio for DKD in men with T2DM. Real-world EMR databases, characterized by continuous sampling, comprehensive clinical variables, and accurate reflection of routine clinical settings, provide an unparalleled opportunity to uncover novel prognostic links between conventional clinical indicators and complex chronic disease outcomes (21). Against this backdrop, leveraging an EMR database from a tertiary care center to conduct a real-world cross-sectional analysis investigating the association between the FPSA/TPSA ratio, DKD risk, and eGFR dynamics holds substantial clinical and translational significance.

Therefore, this study aimed to use a high-quality real-world EMR database to comprehensively evaluate the association between the serum FPSA/TPSA ratio and the risk of DKD in male patients with T2DM. We hypothesized that a significant inverse association exists between the FPSA/TPSA ratio and renal function (eGFR) in this population, and that this association remains robust even after rigorous adjustment for confounders, including age, hypertension, cardiovascular disease, glycemic control, inflammation, and lipid profiles. The findings of this study are expected to provide novel clinical insights and an innovative, non-invasive biomarker strategy for the early identification and risk stratification of DKD.

## 2 Materials and methods

### 2.1 Study design and data source

This study was designed as a single-center, retrospective, cross-sectional analysis. All research data were systematically extracted from the Electronic Medical Record (EMR) database of a tertiary hospital. We retrospectively reviewed the clinical data of patients with T2DM who visited the Department of Endocrinology and related departments between May 2019 and May 2024. The implementation of this study protocol strictly adhered to the ethical principles outlined in the Declaration of Helsinki and was reviewed and approved by the Medical Ethics Committee of Zibo Central Hospital. Given the retrospective nature of this study, which relied on pre-existing EMR data, all personally identifiable patient information was strictly anonymized and de-identified prior to data extraction and analysis. Consequently, the requirement for informed consent was waived by the ethics committee.

### 2.2 Study population

The initial study population comprised male patients with a definitive diagnosis of T2DM. The diagnosis of T2DM was established according to the current criteria of the World Health Organization (WHO) or the American Diabetes Association (ADA). To rigorously minimize potential confounding biases that could influence serum prostate-specific antigen (PSA) levels or obscure renal function assessment, strict inclusion and exclusion criteria were applied.

#### 2.2.1 Inclusion criteria

1. Male patients with a definitive diagnosis of T2DM.
2. Age ≥ 40 years.
3. Availability of complete baseline biochemical data, including TPSA, FPSA, and pertinent renal function indices.

#### 2.2.2 Exclusion criteria

(1) Duplicate medical records (only data from the initial visit and corresponding laboratory tests were retained); (2) A history of acute prostatitis, prostate cancer, or other malignancies involving the urinary or reproductive systems; (3) Baseline TPSA > 10 ng/mL, severe hepatic dysfunction, defined as serum alanine aminotransferase (ALT) or aspartate aminotransferase (AST) levels exceeding three times the upper limit of normal (> 3 × ULN), or explicit EMR documentation of decompensated cirrhosis, acute or chronic liver failure, or severe hepatitis;(4) End-stage renal disease (ESRD), defined as eGFR < 15 mL/min/1.73m^2^, or a documented history of renal replacement therapy (dialysis);(5) Presence of active infection or acute inflammatory status, indicated by a white blood cell (WBC) count > 10 × 10^9^/L;(6) Missing core clinical data, data entry errors, or the presence of extreme outliers.

**Figure 1:**
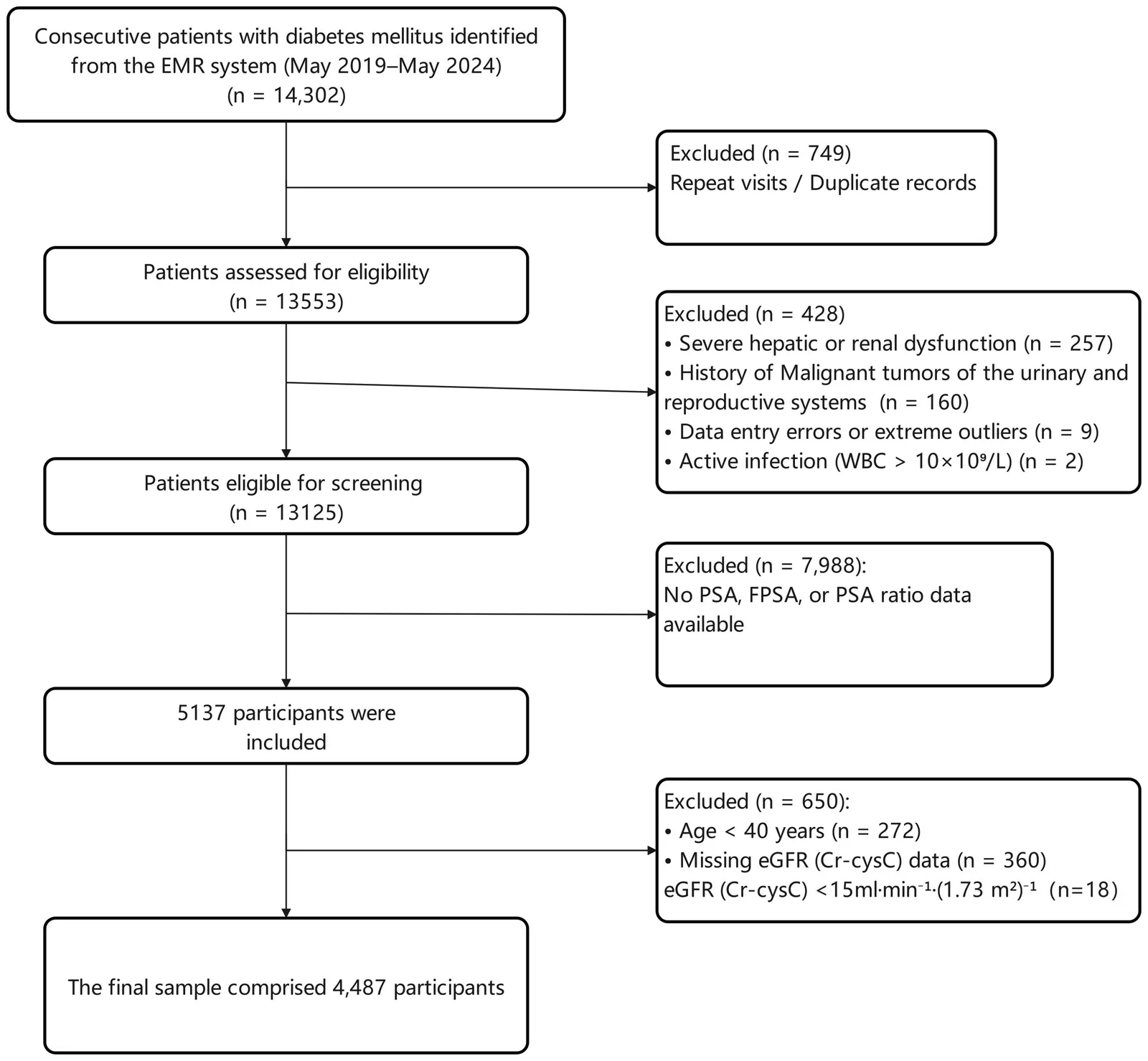
Flowchart of the study population selection process. The diagram illustrates the step-by-step inclusion and exclusion of patients from the initial extraction of electronic medical records (EMR) to the final study sample. Patients were excluded based on strictly defined criteria to minimize confounding factors affecting PSA levels or renal function assessment. DKD, diabetic kidney disease; FPSA, free prostate-specific antigen; TPSA, total prostate-specific antigen; eGFR, estimated glomerular filtration rate; WBC, white blood cell.

Following this rigorous screening process, a total of 4,487 eligible male patients with T2DM were ultimately enrolled in the final statistical analysis cohort (the detailed screening flowchart is delineated in **Figure 1**).

### 2.3 Data collection and variable definitions

#### 2.3.1 Exposure variable

The primary exposure variable in this study was the FPSA/TPSA ratio. Fasting venous blood samples were collected from all patients in the early morning. Serum total prostate-specific antigen (TPSA) and free prostate-specific antigen (FPSA) levels were measured via chemiluminescent immunoassay (CLIA) using an automated immunology analyzer (UniCel DxI 800; Beckman Coulter, Brea, CA, USA). Routine clinical chemistry parameters, including serum creatinine, cystatin C, lipid profiles, and other biochemical markers, were analyzed using an automated clinical chemistry analyzer (Cobas c702; Roche Diagnostics, Mannheim, Germany).The FPSA/TPSA ratio was calculated by dividing the measured FPSA by TPSA and was expressed as a percentage (%). In the statistical analysis, this ratio was modeled both as a continuous variable and as a categorical variable divided into quartiles (Q1–Q4).

#### 2.3.2 Outcome variable

The primary outcome variable was eGFR. To ensure a highly precise assessment of renal function in diabetic patients and to overcome the limitations of creatinine-only formulas, eGFR was calculated using the Chronic Kidney Disease Epidemiology Collaboration (CKD-EPI) equation, combining serum creatinine and cystatin C (CKD-EPI Cr-CysC equation). In accordance with the Kidney Disease: Improving Global Outcomes (KDIGO) guidelines, patients were stratified into three renal function tiers based on eGFR levels: the normal renal function group (eGFR ≥ 90 mL/min/1.73m^2^), the mildly impaired renal function group (60 ≤eGFR < 90 mL/min/1.73m^2^), and the moderate-to-severely impaired renal function group (15 ≤ eGFR < 60 mL/min/1.73m^2^) (22).

#### 2.3.3 Covariates

Guided by existing literature and clinical rationale, baseline covariates potentially influencing PSA metabolism and renal function were systematically extracted from the EMR system, including:

##### 2.3.3.1 Demographics and comorbidities

Age, history of hypertension, and history of cardiovascular disease (CVD).

##### 2.3.3.2 Laboratory biochemical parameters

Fasting blood glucose (FBG), C-reactive protein (CRP), platelet count (PLT), triglycerides (TG), low-density lipoprotein cholesterol (LDL-C), high-density lipoprotein cholesterol (HDL-C), and serum albumin (ALB). All laboratory indices were derived from the results of the initial fasting blood draw upon hospital admission or during the first outpatient visit(Cobas c702; Roche Diagnostics, Mannheim, Germany).

### 2.4 Statistical analysis

The assumption of normality for continuous variables was assessed using the Kolmogorov-Smirnov test, supplemented by visual inspection of histograms and normal quantile-quantile (Q-Q) plots. Normally distributed continuous variables were expressed as mean ± standard deviation (SD), and between-group differences were evaluated using one-way analysis of variance (ANOVA). As the majority of continuous variables in our dataset exhibited skewed distributions, they were presented as medians with interquartile ranges (IQR) and compared across groups using the non-parametric Kruskal-Wallis H test. Categorical variables were summarized as frequencies and proportions (n, %), with group comparisons performed using the Pearson Chi-square test. Statistical analyses were performed using the R software (available at http://www.R-project.org, The R Foundation) and Free Statistics software, version 2.0. and a two-sided P-value < 0.05 was considered statistically significant.

To evaluate the independent association between the FPSA/TPSA ratio and eGFR, multivariable linear regression analyses were performed using three stepwise adjusted models. Model I (Crude Model) was unadjusted for any covariates. Model II (Core Clinical Model) was adjusted for demographic and fundamental clinical variables, including age, body mass index (BMI), systolic blood pressure (SBP), and duration of diabetes. Model III (Fully Adjusted Model) was further adjusted for key metabolic confounders based on Model II, including HbA1c, HDL-C, LDL-C, and uric acid (UA).

The selection of covariates for the fully adjusted model was rigorously conducted to include true baseline confounders while avoiding over-adjustment bias. Notably, highly correlated renal and nutritional parameters were deliberately excluded to prevent the adjustment of variables on the causal pathway. Furthermore, to ensure the mathematical stability of the multivariable models, rigorous collinearity diagnostics were performed prior to model finalization. Variance inflation factors (VIFs) were calculated for all covariates, with a predefined threshold of VIF < 5 indicating the absence of significant multicollinearity. All variables ultimately included in Model III demonstrated VIF values < 5, confirming the reliability of the regression estimates. Regression coefficients (β) and their 95% confidence intervals (95% CIs) were calculated, and a linear trend test (P for trend) was performed. Furthermore, to explore any potential non-linear or dose-response relationship between the FPSA/TPSA ratio and eGFR, we employed restricted cubic splines (RCS) with four knots, superimposed on the fully adjusted model (Model III). Finally, to verify the robustness of the results and identify potential effect modifiers, stratified subgroup analyses were conducted based on age (< 65 years vs. ≥ 65 years), hypertension status, cardiovascular disease status, and FBG levels (< 7.0 mmol/L vs. ≥ 7.0 mmol/L). Statistical analyses were carried out using R software (version 4.2.2, http://www.R-project.org, The R Foundation) and Free Statistics software, version 2.3. The significance of multiplicative interaction terms was assessed to calculate the P for interaction. A two-sided P-value < 0.05 was considered statistically significant.

## 3 Results

### 3.1 Study population screening and enrollment

Using our institution’s EMR database, we initially retrieved records of 14,302 male patients with a confirmed diagnosis of diabetes who visited between May 2019 and May 2024.

Initially, 749 duplicate medical encounters or redundant entries were excluded, leaving 13,553 patients’ primary visit records for subsequent evaluation. To rigorously minimize potential biases, strict inclusion and exclusion criteria were applied, leading to the exclusion of an additional 428 patients for the following reasons: (1) severe hepatic or renal dysfunction (n=257); (2) a documented history of prostate cancer (n=160); (3) data entry errors or extreme outliers (n=9); and (4) active infection indicated by a white blood cell (WBC) count > 10 × 10^9^/L (n=2).

Among the remaining 13,125 eligible candidates, 7,988 were excluded due to the absence of serum PSA profiles (including TPSA, FPSA, or the PSA ratio). Subsequently, 5,137 participants advanced to the final screening phase. In accordance with our predefined criteria, a further 650 individuals were excluded: age under 40 years (n=272), missing eGFR data calculated using the Cr-CysC equation (n=360), and the presence of end-stage renal disease (ESRD) with an eGFR < 15 mL/min/1.73m^2^ (n=18). Ultimately, a well-defined cohort of 4,487 male patients with T2DM was enrolled in the final cross-sectional analysis (**Figure 1**).

### 3.2 Baseline characteristics

The final cohort comprised 4,487 eligible male patients with T2DM. Based on eGFR calculated by the CKD-EPI (Cr-CysC) equation, the patients were stratified into three distinct renal function categories: the normal renal function group (eGFR ≥ 90 mL/min/1.73m^2^, n=1,573), the mildly impaired group (60 ≤eGFR < 90 mL/min/1.73m^2^, n=1,716), and the moderate-to-severely impaired group (15 ≤ eGFR < 60 mL/min/1.73m^2^, n=1,198). The detailed demographic characteristics, clinical parameters, and laboratory findings across these groups are summarized in **Table 1**.

**Table 1:** Baseline characteristics of the study population stratified by eGFR^a^ categories.

Variables	Total (n = 4487)	15≤eGFR <60 (n = 1198)	60≤eGFR <90 (n = 1716)	eGFR ≥90 (n = 1573)	*p*
Age, (years, Mean ± SD)	64.8 ± 11.3	71.8 ± 7.1	63.6 ± 11.9	60.8 ± 10.8	< 0.001
Hypertension n (%)					< 0.001
No	1687 (40.9)	385 (34.9)	657 (41.7)	645 (44.5)	
Yes	2441 (59.1)	719 (65.1)	918 (58.3)	804 (55.5)	
Cardiovascular Disease n (%)					< 0.001
No	2375 (67.8)	502 (55.9)	937 (69.9)	936 (74)	
Yes	1128 (32.2)	396 (44.1)	403 (30.1)	329 (26)	
FBG ^b^(mmol/L,Mean ± SD)	7.9 ± 3.5	7.9 ± 3.7	7.8 ± 3.2	8.1 ± 3.7	0.063
ALB^c^ (g/L, Mean ± SD)	40.3 ± 9.7	39.6 ± 9.1	40.5 ± 9.8	40.5 ± 9.9	0.018
CRP^d^ (mg/L), Median (IQR)	2.9 (0.2, 26.1)	3.2 (0.3, 25.8)	2.8 (0.2, 24.9)	2.8 (0.2, 27.3)	0.409
PLT^e^(*10^9/L), Mean ± SD	204.7 ± 75.7	203.5 ± 75.0	202.8 ± 73.4	207.7 ± 78.6	0.184
TG^f^ (mmol/L), Median (IQR)	1.2 (0.9, 1.7)	1.2 (0.9, 1.7)	1.2 (0.9, 1.7)	1.2 (0.9, 1.7)	0.759
LDL^g^ (mmol/L, Mean ± SD)	2.6 ± 1.0	2.6 ± 1.0	2.6 ± 1.0	2.6 ± 1.0	0.978
HDL^h^ (mmol/L, Mean ± SD)	1.0 ± 0.3	1.0 ± 0.3	1.0 ± 0.3	1.0 ± 0.3	0.951
TPSA^i^(ng/mL), Median (IQR)	0.9 (0.5, 2.0)	0.9 (0.5, 2.0)	0.9 (0.5, 2.1)	0.9 (0.5, 1.9)	0.524
FPSA^j^ (ng/mL), Median (IQR)	0.3 (0.1, 0.5)	0.3 (0.2, 0.6)	0.3 (0.1, 0.5)	0.2 (0.1, 0.4)	< 0.001
FPSA/TPSA(%), Mean ± SD	30.1 ± 14.5	33.2 ± 16.1	29.7 ± 13.7	28.2 ± 13.7	< 0.001

Patients were stratified based on their estimated glomerular filtration rate (eGFR) calculated using the CKD-EPI equation based on combined serum creatinine and cystatin C. Groups are defined as: Normal renal function (eGFR≥90 mL/min/1.73m^2^), Mildly reduced renal function (eGFR 60–89 mL/min/1.73m^2^), and Moderate-to-severely reduced renal function (15≤eGFR < 60 mL/min/1.73m^2^). eGFR^a^, estimated glomerular filtration rate; FBG^b^, Fasting blood glucose; ALB^c^, Albumin; CRP^d^, C-reactive protein; PLT^e^, Platelet; TG^f^, Triglyceride; LDL^g^, Low-density lipoprotein; HDL^h^, high-density lipoprotein; TPSA^i^, Total prostate specific antigen; FPSA^j^, free prostate specific antigen.

Notably, PSA profiles exhibited a striking divergence in distribution patterns across different renal functional states. While TPSA levels remained remarkably stable across the three groups, with no statistically significant difference (P = 0.524), FPSA levels increased significantly in parallel with the decline in eGFR (P<0.001). This kinetic discrepancy culminated in a highly significant, stepwise increase in the serum FPSA/TPSA ratio across the strata: 28.2 ± 13.7 in the normal group, 29.7 ± 13.7 in the mildly impaired group, and 33.2 ± 16.1 in the moderate-to-severely impaired group (P<0.001).

Regarding demographics and baseline comorbidities, worsening renal impairment was accompanied by a significantly higher mean age (increasing from 60.8 to 71.8 years, P<0.001). Correspondingly, the prevalence of hypertension (55.5% vs. 58.3% vs. 65.1%, P<0.001) and a history of cardiovascular disease (26.0% vs. 30.1% vs. 44.1%, P<0.001) was markedly higher in the lower eGFR tiers. In terms of nutritional and metabolic indices, serum albumin (ALB) levels demonstrated a significant decline in the cohort with the most severe renal impairment (P = 0.018). Interestingly, traditional metabolic and inflammatory markers—including FBG, CRP, PLT, and lipid profiles (TG, LDL-C, HDL-C)—did not show statistically significant differences across the three renal function categories (all P>0.05).

### 3.3 Association between the FPSA/TPSA ratio and eGFR

Univariate linear regression analysis revealed a significant inverse association between the FPSA/TPSA ratio and eGFR (β = −0.20, 95% CI: -0.25 to -0.15, P<0.001). Age was also strongly and negatively correlated with eGFR (β = −0.85, 95% CI: -0.91 to -0.79, P<0.001). Furthermore, TG exhibited a significant positive correlation with eGFR (β = 0.51, 95% CI: 0.09 to 0.94, P = 0.018). In contrast, hypertension, cardiovascular disease, CRP, HDL-C, PLT, FBG, and ALB showed no statistically significant associations with eGFR in the univariate model (all P>0.05).(**Table 1**).

**Figure 2:**
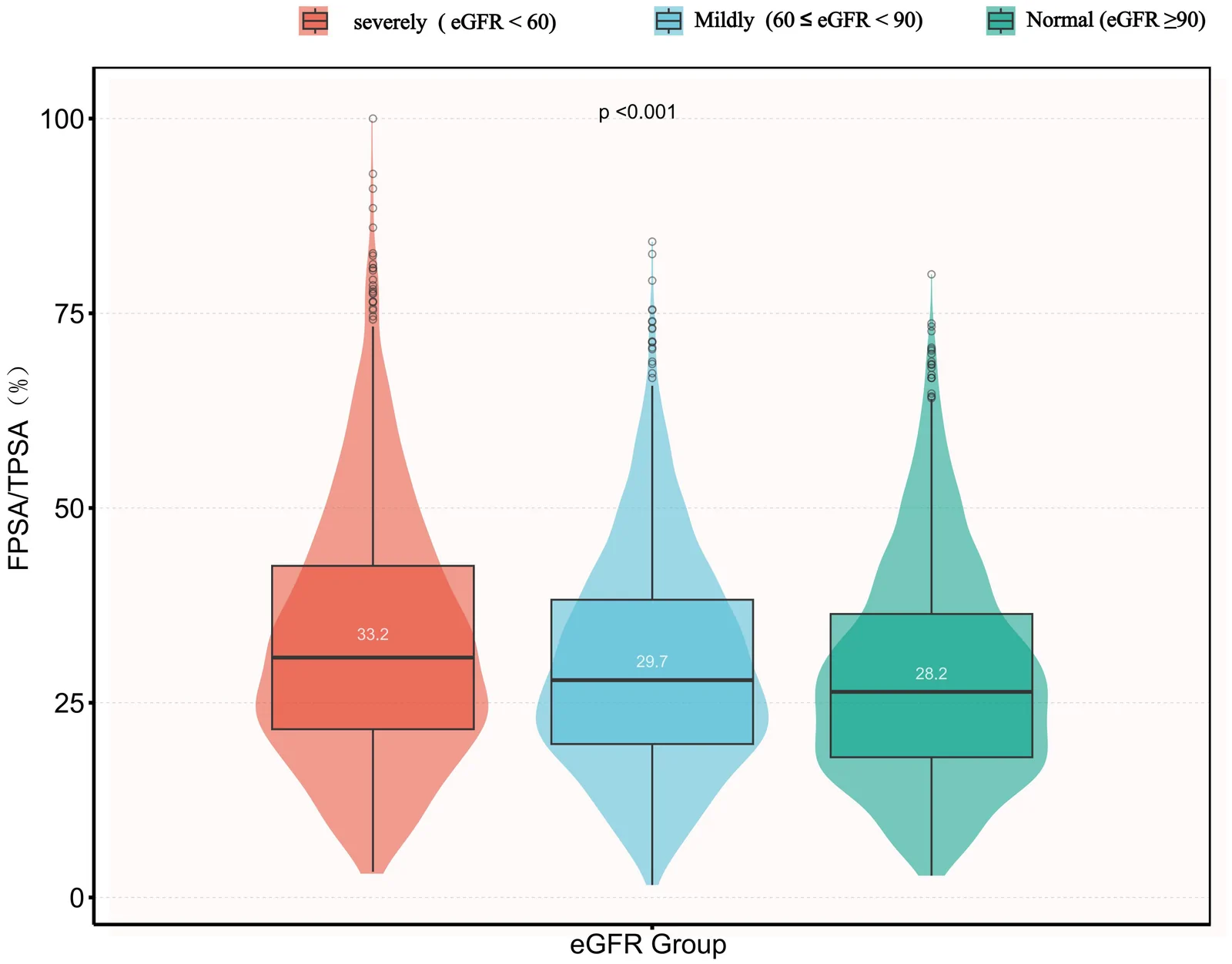
Distribution of FPSA/TPSA ratio across different eGFR categories. The violin plots illustrate the probability density distribution of the FPSA/TPSA ratio in male patients with T2DM, stratified by renal function: moderate-to-severely impaired (15 ≤eGFR < 60 mL/min/1.73m^2^, red), mildly impaired (60 ≤ eGFR < 90 mL/min/1.73m^2^, blue), and normal renal function (eGFR ≥ 90 mL/min/1.73m^2^, green). Nested within each violin, the box plots display the interquartile range (IQR), with the bold horizontal line indicating the median. The annotated values (33.2, 29.7, and 28.2) represent the mean ratio for each respective group. Points extending beyond the whiskers denote outlier values. The global P-value (< 0.001), determined by the Kruskal-Wallis test, indicates highly significant differences across the three functional strata.

To visually depict the impact of declining renal function on FPSA-related indices, we generated violin plots overlaid with box plots to illustrate the central tendency and data distribution of the FPSA/TPSA ratio across different eGFR groups (**Figure 2**). As illustrated, the overall distribution profile of the FPSA/TPSA ratio exhibited a pronounced upward and rightward shift as the severity of renal impairment progressed (global P<0.001). From the normal renal function group (green, mean 28.2) to the mildly impaired group (blue, mean 29.7) and the moderate-to-severely impaired group (red, mean 33.2), the overall baseline of the box plots (medians and IQRs) demonstrated a clear, stepwise ascending trajectory.

To further rigorously evaluate the independent association and dose-response relationship between the FPSA/TPSA ratio and eGFR, three stepwise adjusted multivariable linear regression models were constructed, analyzing the ratio both as a continuous variable and as quartiles (Q1–Q4) (**Table 2**). When modeled continuously, the unadjusted crude model (Model I) indicated a significant negative correlation between the FPSA/TPSA ratio and eGFR (β = −0.25, 95% CI: -0.30 to -0.20, P<0.001). Upon adjustment for age, hypertension, and CVD (Model II), this inverse association remained highly significant (β = −0.18, P<0.001). Even in the most stringent Fully Adjusted Model (Model III)—which further accounted for potential confounding by glycemic control (FBG), inflammation (CRP), lipid profiles (LDL-C, HDL-C), and PLT—each 1-unit increment in the FPSA/TPSA ratio independently predicted a 0.14 mL/min/1.73m^2^ decline in eGFR (95% CI: -0.21 to -0.07, P<0.001).

**Table 2:** Association of FPSA/TPSA with eGFR.

Categories	^a^Model I	*p*	^b^Model II	*p*	Model ^c^III	*p*
	β (95% CI)		β (95% CI)		β (95% CI)	
FPSA/TPSA (%)						
Q1(1.6-19.4)	Ref		Ref		Ref	
Q2(19.5-27.9)	-4.22 (-6.32~-2.12)	<0.001	-3.52 (-5.74~-1.3)	0.002	-3.78 (-6.5~-1.07)	0.006
Q3(28-38.7)	-4.97 (-7.07~-2.87)	<0.001	-2.89 (-5.13~-0.66)	0.011	-2.28 (-5.04~0.48)	0.106
Q4(38.8-100)	-8.94 (-11.04~-6.84)	<0.001	-6.62 (-8.86~-4.38)	<0.001	-6.05 (-8.8~-3.3)	<0.001
*p*-value for trend		<0.001		<0.001		<0.001
Continuous	-0.25 (-0.3~-0.2)	<0.001	-0.18 (-0.23~-0.12)	<0.001	-0.14 (-0.21~-0.07)	<0.001

Data are presented as regression coefficients (β) and 95% confidence intervals (CI). The FPSA/TPSA was categorized into quartiles (Q1-Q4), with the lowest quartile (Q1) serving as the reference group. Multivariable linear regression models were used to evaluate the association between FPSA/TPSA ratio and eGFR levels.

a Model I: no adjusted.

b Model II: adjusted for age + Hypertension +CVD.

c Model III: adjusted for age + Hypertension +CVD +Fasting blood glucose(FBG)+C-reactive protein(CRP) + Platelet (PLT)+Low-density lipoprotein(LDL)+high-density lipoprotein(HDL).

eGFR, estimated glomerular filtration rate; FPSA, free prostate-specific antigen; TPSA, total prostate-specific antigen; Ref, reference; CI, confidence interval. Collinearity diagnostics confirmed that no substantial multicollinearity existed among the included covariates in the fully adjusted model, with all VIF values well below the stringent threshold of 5 (ranging from1.08 to1.31, ensuring the stability of our regression coefficients.

When the FPSA/TPSA ratio was analyzed as categorical quartiles, a striking progressive decline in renal function was observed. Using the lowest quartile (Q1, ratio: 1.6–19.4) as the reference baseline, progression across the quartiles corresponded to a progressive deterioration in eGFR levels. In the comprehensively adjusted Model III, patients in the highest quartile (Q4, ratio: 38.8–100) exhibited a substantial and significant reduction in eGFR compared with the Q1 cohort (β = −6.05, 95% CI: -8.80 to -3.30, P<0.001). Although the statistical significance for the Q3 group was slightly attenuated in Model III (P = 0.106), the entire cohort maintained a highly significant linear downward trend across all three models (all P for trend < 0.001).

### 3.4 Restricted cubic spline and dose-response analysis

**Figure 3:**
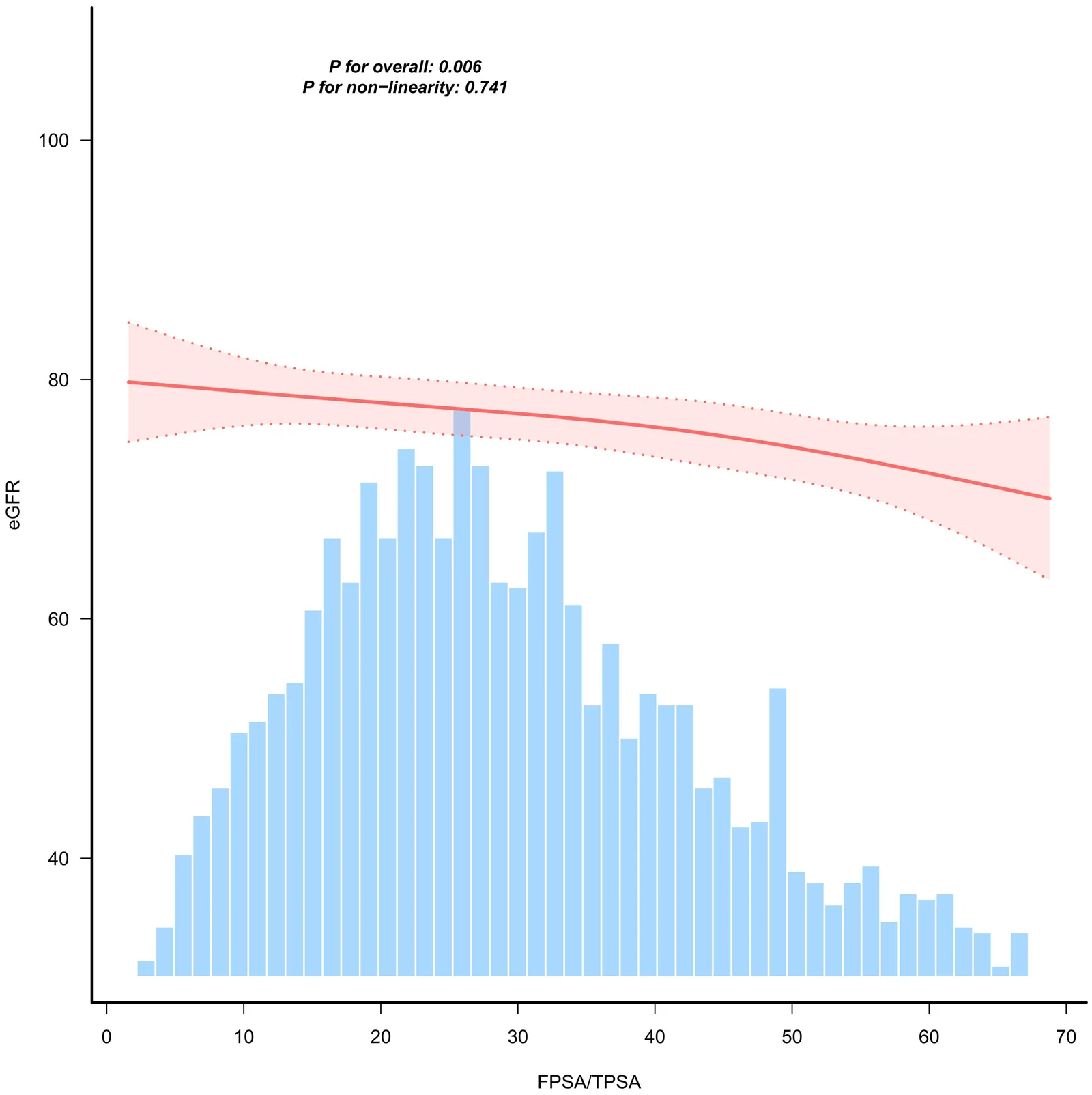
Restricted cubic spline (RCS) plot for the dose-response relationship between FPSA/TPSA ratio and eGFR. The solid red line represents the fitted predicted values of eGFR, while the pink shaded area denotes the 95% confidence intervals (CI). The light blue histogram at the bottom visualizes the frequency distribution of the FPSA/TPSA ratio within the study population. The model was fully adjusted for covariates included in Model III (age, hypertension, cardiovascular disease, FBG, CRP, PLT, LDL-C, and HDL-C). The overall association was statistically significant (P for overall = 0.006), and the non-linearity test confirmed a strict linear inverse relationship (P for non-linearity = 0.741).

To further delineate any potential non-linear relationships or threshold effects between the FPSA/TPSA ratio and eGFR, we applied RCS dose-response analysis superimposed on the fully adjusted model (Model III) (**Figure 3**).

The RCS analysis confirmed a significant overall association between the FPSA/TPSA ratio and eGFR (P = 0.006). Crucially, the test for non-linearity revealed that this relationship lacked complex non-linear inflection points (P for non-linearity = 0.741, well above the 0.05 significance threshold). As depicted by the red trend line in **Figure 3**, across the entire distribution spectrum of the data (contextualized by the bottom frequency histogram), a progressive elevation in the serum FPSA/TPSA ratio was consistently associated with a steady, smooth, and strictly linear decline in eGFR.

### 3.5 Subgroup analyses and interaction tests: robustness of the association

**Figure 4:**
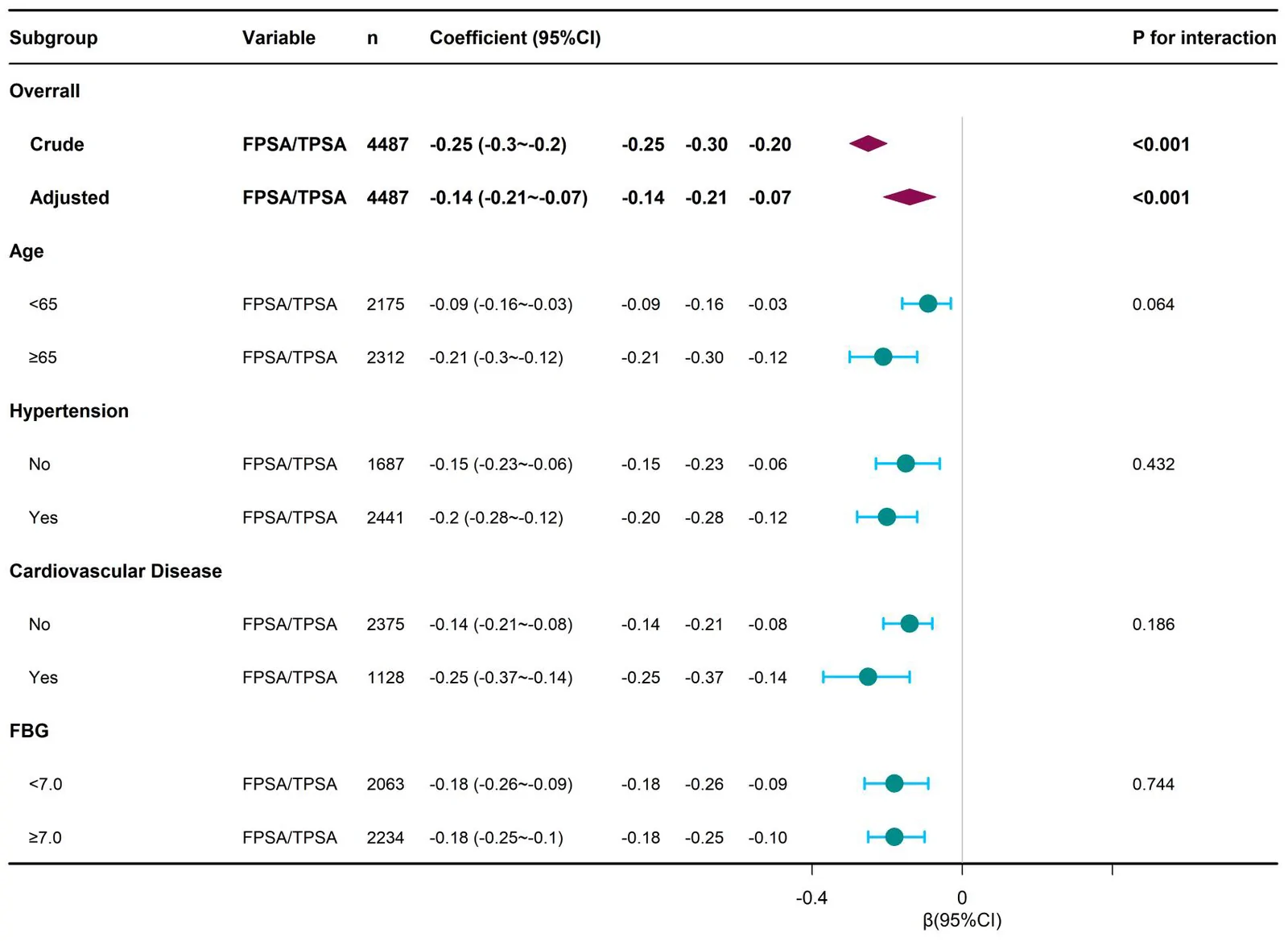
Forest plot of subgroup analyses assessing the association between the FPSA/TPSA ratio (%) and eGFR. The forest plot depicts the β coefficients (solid points/diamonds) and their 95% confidence intervals (horizontal bars) for the association between the FPSA/TPSA ratio and eGFR, stratified by key clinical variables. All models were adjusted for age, hypertension, cardiovascular disease, FBG, CRP, PLT, LDL-C, and HDL-C, except for the stratifying variable itself in each respective subgroup. The P for interaction was calculated to evaluate the consistency of the association across different sub-cohorts. CI, confidence interval; FBG, fasting blood glucose.

To validate the robustness of the inverse correlation between the FPSA/TPSA ratio (%) and eGFR, and to identify any potential effect modifiers, we conducted stratified subgroup analyses based on age, history of hypertension, history of CVD, and FBG levels (**Figure 4**).

The subgroup analyses demonstrated that the inverse association between the FPSA/TPSA ratio and eGFR remained highly consistent across all evaluated subgroups. Whether stratified by age (< 65 years vs. ≥ 65 years), presence or absence of hypertension, presence or absence of CVD, or glycemic control levels (FBG < 7.0 vs. ≥ 7.0 mmol/L), the β coefficients consistently remained in the negative range and retained statistical significance (none of the 95% CIs crossed the zero line). Furthermore, interaction tests unequivocally confirmed that these common clinical variables did not significantly modify or confound the primary association between the FPSA/TPSA ratio and eGFR (all P for interaction > 0.05).

## 4 Discussion

Drawing upon a high-quality real-world data (RWD) cohort, the present study comprehensively explored the association between the serum FPSA/TPSA ratio and the risk of diabetic kidney disease (DKD) in male patients with T2DM. Our principal findings demonstrate that an elevated serum FPSA/TPSA ratio (%) is significantly and independently associated with a decline in estimated glomerular filtration rate (eGFR). This inverse correlation remained highly robust even after rigorous adjustment for age, prostate-specific pathologies, and metabolic confounders, exhibiting remarkable consistency across diverse clinical subgroups.

When interpreting an elevated FPSA/TPSA ratio in the context of renal impairment, it is imperative first to exclude its traditional clinical implications related to prostate cancer or BPH screening (23, 24). In typical prostate pathology, such as prostate cancer, the proportion of complexed PSA increases, leading to a decrease in the FPSA/TPSA ratio. Conversely, our study observed an increase in this ratio as renal function declined. The demographic characteristics of our cohort (**Table 1**) provide compelling evidence in this regard: the median TPSA levels remained consistently stable at approximately 0.9 ng/mL across varying eGFR strata, with no statistically significant difference observed (P = 0.524). Furthermore, by strictly excluding patients with a history of prostate cancer, acute prostatitis, or markedly elevated baseline TPSA levels (>10ng/mL), we minimized the confounding effects of subclinical prostate pathology. This underscores that the structural health of the prostate in our cohort was largely homogeneous, and that patients did not harbor more severe prostate lesions simply because of the presence of DKD.

The underlying mechanism of this “phenotypic divergence “lies in the distinct metabolic kinetics of FPSA and TPSA. In the bloodstream, TPSA predominantly binds to α1-antichymotrypsin (ACT) or α2-macroglobulin (A2M), forming large complexes (approximately 100 kDa) that are primarily metabolized by the hepatic system (2, 25). Conversely, FPSA exists as a smaller molecule (approximately 30 kDa), bypasses hepatic metabolism, and relies almost exclusively on glomerular filtration for urinary excretion (3). Because of its low molecular weight, FPSA functions physiologically similarly to other low-molecular-weight proteins (such as β2-microglobulin) in the context of renal filtration (26, 27). Consequently, when patients with T2DM experience renal microvascular damage and a subsequent decline in eGFR, the hepatic clearance of TPSA remains unaffected. In contrast, the renal excretion of FPSA is compromised, leading to its systemic accumulation. This pharmacokinetic trade-off establishes a complete pathophysiological loop explaining the markedly elevated peripheral FPSA/TPSA ratio in patients with DKD, confirming that variations in this ratio in our cohort reflect impaired renal filtration capacity rather than subclinical prostate disease (28, 29). It is important to note that current evidence suggests the elevated FPSA/TPSA ratio acts primarily as a secondary biomarker reflecting altered renal clearance, rather than a direct pathogenic driver of kidney decline. However, to fully elucidate this relationship, future research is warranted. Specifically, longitudinal cohort studies are needed to map the temporal trajectory of the FPSA/TPSA ratio against eGFR decline and albuminuria progression to validate its early predictive value. Additionally, basic experimental studies should be conducted to definitively rule out any direct nephrotoxic or profibrotic effects of FPSA on renal cells.

In terms of effect size evaluation, the multivariable model showed that for every 1% increase in the FPSA/TPSA ratio, eGFR decreased significantly by 0.2 mL/min/1.73m^2^(β = −0.2, P < 0.001), indicating substantial clinical utility. It is worth noting that the negative coefficient of age (β = −0.85) in the model was larger than that of the FPSA/TPSA ratio. From a pathophysiological perspective, this is an expected phenomenon, as age is universally recognized as the primary determinant driving physiological renal senescence (30). Furthermore, the mathematical architecture of the CKD-EPI equation inherently assigns substantial weight to age (31). However, the pivotal value of our finding is that, after robustly accounting for the dominant confounding effect of age, the FPSA/TPSA ratio remained highly statistically significant (P < 0.001). This unequivocally establishes the ratio not merely as a bystander of aging, but as an independent and robust biomarker reflecting DKD-specific impairment.

To ensure the precision and contemporaneity of our real-world findings, we did not rely on serum creatinine (Cr) alone for eGFR estimation. Instead, we adopted the CKD-EPI Cr-CysC combined equation (eGFR-Cr-CysC) recommended by the latest KDIGO guidelines (32, 33). Recent high-quality clinical trials have corroborated that, because serum creatinine is susceptible to variations in muscle mass and nutritional status, whereas cystatin C (CysC) can be influenced by inflammation and thyroid function, their combination represents the current non-radionuclide gold standard for renal assessment (34, 35). The use of this high-fidelity metric substantially strengthens the evidence linking the FPSA/TPSA ratio to actual renal function.

In analyzing metabolic covariates, we observed two phenomena that might appear to contradict conventional cardiovascular risk paradigms but are deeply rooted in cCKD pathology. First, TG exhibited a positive correlation with eGFR (β = 0.51, P = 0.018). Rather than implying a protective effect of hypertriglyceridemia, we postulate that this reflects reverse causality. This paradox—whereby higher lipid levels correlate with better renal outcomes—is the classic “lipid paradox” or reverse epidemiology recognized in advanced CKD populations (11, 36, 37). As renal function deteriorates, patients frequently develop “malnutrition-inflammation-atherosclerosis (MIA) syndrome” (38), which represents a severe state of malnutrition associated with advanced CKD, leading to systemic depletion and unexpectedly low lipid profiles. Conversely, maintaining relatively higher TG levels may indicate a preserved systemic nutritional state (39). Furthermore, this association may also partially reflect residual confounding. Patients with more advanced diabetic kidney disease (lower eGFR) are more likely to be subjected to strict dietary restrictions or intensive lipid-lowering therapies (e.g., statins or fibrates) as part of their cardiovascular risk management (36, 40).Therefore, the lower TG levels observed in these patients are likely a consequence (reverse causality) of both malnutrition-driven health deterioration and intensive clinical management, rather than a protective physiological mechanism.Second, FBG lacked statistical significance in our model (*P* = 0.934). This is because a single FBG measurement merely captures a transient metabolic snapshot, which is highly vulnerable to recent diet, stress, and medication half-lives (41). In contrast, DKD results from long-term cumulative glucotoxicity, rendering time-weighted average exposure indices such as glycated hemoglobin (HbA1c) far more reliable for assessing the risk of microvascular complications (14, 42).

To identify potential effect modifiers and validate the generalizability of our findings, we performed stratified subgroup analyses based on age, history of hypertension, history of CVD, and FBG levels (**Figure 4**). Encouragingly, the inverse association between the FPSA/TPSA ratio and eGFR remained highly consistent across all strata, with none of the 95% confidence intervals for the β coefficients crossing zero. Interaction tests further confirmed that these prevalent clinical factors did not significantly attenuate the predictive efficacy of this biomarker (all P for interaction > 0.05). Of particular clinical interest was the steeper effect trend observed in the elderly cohort (≥ 65 years) compared with their younger counterparts (β = −0.21 vs. −0.09; P for interaction = 0.064) (43). Although the interaction narrowly missed the conventional threshold for statistical significance, this trend suggests that older men—who often have longer durations of diabetes and superimposed senile glomerulosclerosis—may have increased renal sensitivity to FPSA filtration blockade, offering a highly sensitive window for early screening in geriatric T2DM populations.

We acknowledge several limitations of the present study. First, given its cross-sectional design, we could establish only a robust association, rather than absolute causality, between the FPSA/TPSA ratio and DKD. Current evidence suggests this ratio acts primarily as a secondary biomarker reflecting altered renal clearance rather than a direct pathogenic driver. Future prospective longitudinal cohorts are required to map the dynamic trajectory of this ratio alongside eGFR decline, supplemented by basic experimental studies to rule out direct nephrotoxicity. Second, constrained by the retrospective nature of real-world data (RWD), precise prostate volume measurements (e.g., via transrectal ultrasound) were not incorporated into our multivariable models. However, the stability of TPSA largely mitigated macroscopic prostatic interference. Third, the generalizability of this biomarker is inherently restricted by biological and clinical factors. Biologically, its utility is strictly limited to male patients due to the origin of PSA. Clinically, while pharmacokinetic principles suggest that FPSA accumulation occurs in general chronic kidney disease regardless of etiology, our cohort exclusively comprised male patients with T2DM; thus, its predictive performance in non-diabetic nephropathies requires further validation. Fourth,because our primary outcome (eGFR) was calculated using the CKD-EPI Cr-CysC equation, we could not statistically compare the FPSA/TPSA ratio head-to-head against Cystatin C as an independent predictor due to inherent mathematical coupling. Furthermore, our retrospective dataset lacked comprehensive data on novel urinary tubular biomarkers (e.g., KIM-1, NGAL), which are necessary for future studies to fully establish the incremental predictive value of this ratio as an adjunctive warning sign rather than a standalone diagnostic criterion. Fifth, regarding the observed TG-eGFR paradox, the lack of standardized nutritional assessments (e.g., Subjective Global Assessment scores) and detailed medication logs in our retrospective dataset prevented us from definitively quantifying the impact of reverse causality and residual confounding. Lastly, as this study was confined to a Chinese population, standardization of the clinical cut-off values for this biomarker in multi-center, multi-ethnic populations remains necessary.

## 5 Conclusion

In conclusion, after adjusting for age and metabolic confounders, an elevated serum FPSA/TPSA ratio is significantly associated with progressive decline in eGFR among male patients with type 2 diabetes mellitus (T2DM). These results suggest that this routinely measured tumor marker may be repurposed as a non-invasive, cost-effective biomarker for the early detection of diabetic kidney disease (DKD). Incorporating the FPSA/TPSA ratio into standard clinical workflows could enhance risk stratification; however, validation in large-scale, prospective cohort studies is warranted to establish evidence-based, standardized diagnostic cut-offs.

## Data Availability

The raw data supporting the conclusions of this article will be made available by the authors, without undue reservation.

## Glossary

A2M: α2-Macroglobulin
ACT: α1-Antichymotrypsin
ADA: American Diabetes Association
ALB: Serum Albumin
ALT: Alanine Aminotransferase
ANOVA: Analysis of Variance
AST: Aspartate Aminotransferase
BPH: Benign Prostatic Hyperplasia
CI: Confidence Interval
CKD: Chronic Kidney Disease
CKD-EPI: Chronic Kidney Disease Epidemiology Collaboration
Cr: Serum Creatinine
CRP: C-Reactive Protein
CysC: Cystatin C
CVD: Cardiovascular Disease
DKD: Diabetic Kidney Disease
eGFR: Estimated Glomerular Filtration Rate
EMR: Electronic Medical Record
ESKD: End-Stage Kidney Disease
ESRD: End-Stage Renal Disease
FBG: Fasting Blood Glucose
FPSA: Free Prostate-Specific Antigen
GLP-1: Glucagon-Like Peptide-1
HbA1c: Glycated Hemoglobin
HDL-C: High-Density Lipoprotein Cholesterol
IQR: Interquartile Range
KDIGO: Kidney Disease: Improving Global Outcomes
LDL-C: Low-Density Lipoprotein Cholesterol
MIA: Malnutrition-Inflammation-Atherosclerosis
PLT: Platelet Count
PSA: Prostate-Specific Antigen
RAS: Renin-Angiotensin System
RCS: Restricted Cubic Spline
RWD: Real-World Data
SD: Standard Deviation
SGLT2: Sodium-Glucose Cotransporter 2
T2DM: Type 2 Diabetes Mellitus
TG: Triglycerides
TPSA: Total Prostate-Specific Antigen
UACR: Urinary Albumin-to-Creatinine Ratio
ULN: Upper Limit of Normal
VIF: Variance Inflation Factor
WBC: White Blood Cell
WHO: World Health Organization
